# Multiple Conjunctival Papillomas of Eyelid Margins in Pemphigus vulgaris

**DOI:** 10.1155/2011/174912

**Published:** 2011-12-21

**Authors:** Inbal Avisar, Iftach Yassur, Israel Kremer

**Affiliations:** ^1^Department of Ophthalmology, Rabin Medical Center, Beilinson Campus, Petah Tiqwa 49100, Israel; ^2^Sackler Faculty of Medicine, Tel Aviv University, Tel Aviv 69978, Israel

## Abstract

Ocular involvement in pemphigus vulgaris (PV) ranges from mild conjuctivits to conjunctival blisters and rarely prominent erosions of the bulbar and palpebral conjunctiva which may be found at the eyelid margin. We report two unusual cases of PV patients presenting with multiple conjunctival papillomatous lesions of the eyelids margins.

## 1. Introduction

The characteristic ocular finding in pemphigus vulgaris (PV) is conjunctivitis with hyperemia, and mucus discharge. Conjunctival blisters, erosions and synechia are rare. We report two unusual cases of PV patients presenting with multiple conjunctival papillomatous lesions of the eyelids margins.

## 2. Case 1

**Figure 1:**
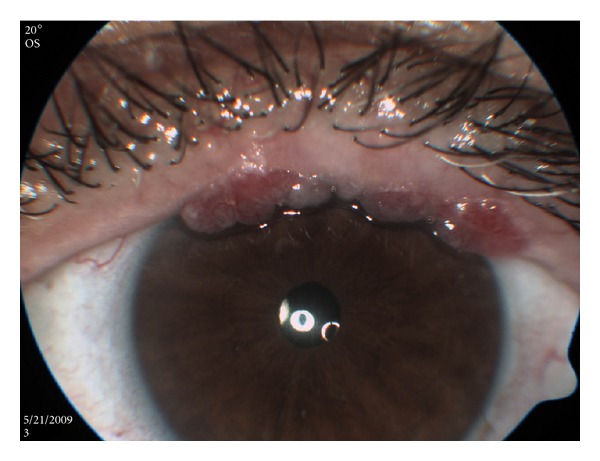


A 53-year-old woman with PV diagnosed according to clinical, histopathologic, and immunological criteria since 1997, presented in the past with oral mucosa, pharyngeal, and esophageal ulcers, without cutaneous involvement. She has been treated with low-dose systemic triamcinolone 4 mg OD and found to be in remission for the last several years. She was referred to our clinic due to symptomatic eyelid margin mucosal papillomas (Figure 1), characteristic of human papilloma virus (HPV). Virologic tests were inconclusive for HPV. She was offered to undergo ablation with cryotherapy but refused because of her known general tendency to infections. 

## 3. Case 2

A 58-years-old woman with diagnosed PV, treated with systemic prednisolone (5 mg OD) and imuran (100 mg OD), presented in the past with oral mucosa and pharyngeal ulcers. She was followed by her dermatologist and found to be in remission for the last several years. She was referred to our clinic because of ocular irritation associated with small multiple mucosal papillomas in the eyelid margin of both eyes mainly in the medial canthal area. The lesions were ablated twice in every eye during the past five years using cryotherapy. However, recurrences were documented several months following each cryo-resection. The histopathologic examination of the biopsied tissue revealed nonspecific neutrophillic and plasma cells infiltration with cuboidal epithelial cells covering the substantia propria of the papilloma. Unfortunately, viral tests were not performed.

## 4. Comment

Ocular involvement in PV ranges from mild conjuctivits to conjunctival blisters and rarely prominent erosions of the bulbar and palpebral conjunctiva which may be found at the eyelid margin. Biopsy of the affected conjunctivae has demonstrated similar histopathologic and direct immune-fluorescent findings to the skin finding of PV [1]. There is probably no correlation between ocular findings and disease severity, which may persist chronically after healing of blistering cutaneous lesions [2].

Squamous cell papillomata of the conjunctiva are among the most common benign lesions of the conjunctiva, located mainly medially and inferiorly. The clinical course favors spontaneous regression cure [3, 4]. In those cases where large lesions cause symptoms or a cosmetic defect, surgery is recommended with cryotherapy to the remaining conjunctiva to prevent recurrence [4]. Previous studies revealed the presence of human papilloma virus in 58–92% of conjunctival papillomas [3].

The association between pemphigus vulgaris and conjunctival papillomata is not documented in the literature [1] and it is questioned in this setting. Furthermore, The association between conjunctival papilloma and immunedeficiency and AIDS has been raised in the past. However, this association was not proved in the literature [5].

Above describes two patients with the finding of multiple conjunctival papillomas of the eyelid margin. Our two described patients were in total remission following systemic steroid therapy. The fact they had eyelid involvement did not correlate with the severity of their immunologic disease.

The biopsy performed in one of these patients revealed only mild reactive changes, including mixed inflammatory cell infiltration with plasma cells. No acantholysis or bullae were observed. We postulate that both presented cases had HPV-related conjunctival papilloma. We state that this presumed diagnosis relies on the clinical appearance of the multiple papillomatous lesions, as the virologic tests were inconclusive. We suspect that the pathogenesis of multiple conjunctival papillomata (probably of viral origin) in these cases is related to the immune-suppressed situation with chronic use of corticosteroids and immunosuppressive therapy.

## References

[1] Hodak E, Kremer I, David M (1990). Conjunctival involvement in pemphigus vulgaris: a clinical, histopathological and immunofluorescence study. *British Journal of Dermatology*.

[2] Palleschi GM, Giomi B, Fabbri P (2007). Ocular involvement in pemphigus. *American Journal of Ophthalmology*.

[3] Verma V, Shen D, Sieving PC, Chan CC (2008). The role of infectous Agents in the etiology of ocular adnexal neoplasia. *Survey of Ophthalmology*.

[4] Shields CL, Shields JA (2004). Tumors of the conjunctiva and cornea. *Survey of Ophthalmology*.

[5] Pantanowitz L, Schlecht HP, Dezube BJ (2006). The growing problem of non-AIDS-defining malignancies in HIV. *Current Opinion in Oncology*.

